# Knowledge of hypertension and understanding of home blood pressure monitoring among hypertensive patients in India: results from the GRAND Study

**DOI:** 10.1038/s41440-026-02763-w

**Published:** 2026-08-21

**Authors:** Anuj Maheshwari, Noriko Matsushita, Rituparna Barooah, Smitha Bhat, Natarajan Bhavatharini, Arun Goel, Aloke Kumar Gupta, Amit Gupta, Arvind Gupta, Vitull Kumar Gupta, Aravinda Jagadeesha, Rachna Parashar, Bijay Patni, Banshi Saboo, Divya Saxena, Nagendra Kumar Singh, Ramji Singh, Ruchi Singh, Saurabh Srivastava, Sachiko Ohde, Yutaka Imai, Narsingh Verma, Takayoshi Ohkubo

**Affiliations:** 1 Department of Internal Medicine, Hind Institute of Medical Sciences, Heerpur, Uttar Pradesh India; 2 Global Medical Affairs, Asia Pacific Regional, Omron Healthcare Singapore, Pte. Ltd., Alexandra Techno Park, Singapore; 3 Graduate School of Public Health, St. Luke’s International University, Tokyo, Japan; 4 Department of Physiology, Northeastern Indira Gandhi Regional Institute of Health and Medical Sciences, Shillong, Meghalaya India; 5 Father Muller Medical College, Kankanady, Mangalore, India; 6 S.R.C Diabetes Care Centre, Erode, Tamil Nadu India; 7 Department of Physiology, All India Institute of Medical Sciences (AIIMS), Rishikesh, Uttarakhand India; 8 Diabetes Education and Research Center, Gorakhpur, Uttar Pradesh India; 9 Centre for Diabetes Care, Greater Noida, Uttar Pradesh India; 10 Department of Internal Medicine and Diabetes, RHL- Rajasthan Hospital, Rajasthan Jaipur, India; 11 Department of Medicine, Kishori Ram Hospital and Diabetes Care Centre, Bathinda, Punjab India; 12 Dr. Arvinda’s Diabetes Centre, Bengaluru, Karnataka India; 13 Department of Physiology, All India Institute of Medical Sciences (AIIMS), Bhopal, Madhya Pradesh India; 14 Diabetes Wellness Care, Kolkata, West Bengal India; 15 Diabetes, Diacare, Ahmedabad, Gujarat India; 16 Saxena Multispeciality Hospital, Sonipat, Haryana India; 17 Department of Medicine, Diabetes and Heart Research Centre, Dhanbad, Jharkhand India; 18 Executive Director, All India Institute of Medical Sciences (AIIMS), Kalyani, West Bengal India; 19 Department of Medicine, Government Institute of Medical Sciences, Greater Noida, Uttar Pradesh India; 20 Tohoku Institute for Management of Blood Pressure, Sendai, Miyagi Japan; 21 Principal and Dean, Hind Institute of Medical Sciences, Lucknow, Uttar Pradesh India; 22 Department of Hygiene and Public Health, Teikyo University School of Medicine, Tokyo, Japan

**Keywords:** Home blood pressure monitoring, Hypertension, India, Patient knowledge, Survey

## Abstract

Home blood pressure monitoring (HBPM) is recommended for the management of hypertension; however, patients’ understanding of hypertension and HBPM remains insufficient. This multicenter cross-sectional study assessed knowledge of hypertension and HBPM among 1920 patients receiving pharmacological treatment across 12 states in India and identified factors associated with relatively higher knowledge. Knowledge was evaluated across four domains: understanding of clinic and home blood pressure (BP) thresholds, awareness of causes of hypertension, recognition of risks associated with uncontrolled BP, and awareness of the benefits of HBPM. The median knowledge score was 1 (range 0–7), indicating generally low knowledge. Knowledge varied across domains, with particularly poor understanding of BP thresholds and limited awareness of HBPM benefits, while awareness of causes and complications was relatively higher but still insufficient. Relatively higher knowledge (≥75th percentile) was observed in 38.0% of participants and was more common among participants with prior HBPM experience than among those without prior HBPM experience (46.9% vs. 29.9%, *p* < 0.001). Higher education, type of healthcare facility, and prior HBPM experience were associated with relatively higher knowledge. These findings suggest that knowledge of hypertension among patients in India is limited and domain-specific, with critical gaps in areas essential for interpreting and utilizing BP measurements. Improving hypertension management may require not only promoting HBPM but also strengthening patient education. Clinical trial registration status: Registered at Clinical Trials Registry-India (CTRI) under CTRI/2023/02/049486 on 06 February 2023.

**Figure:**
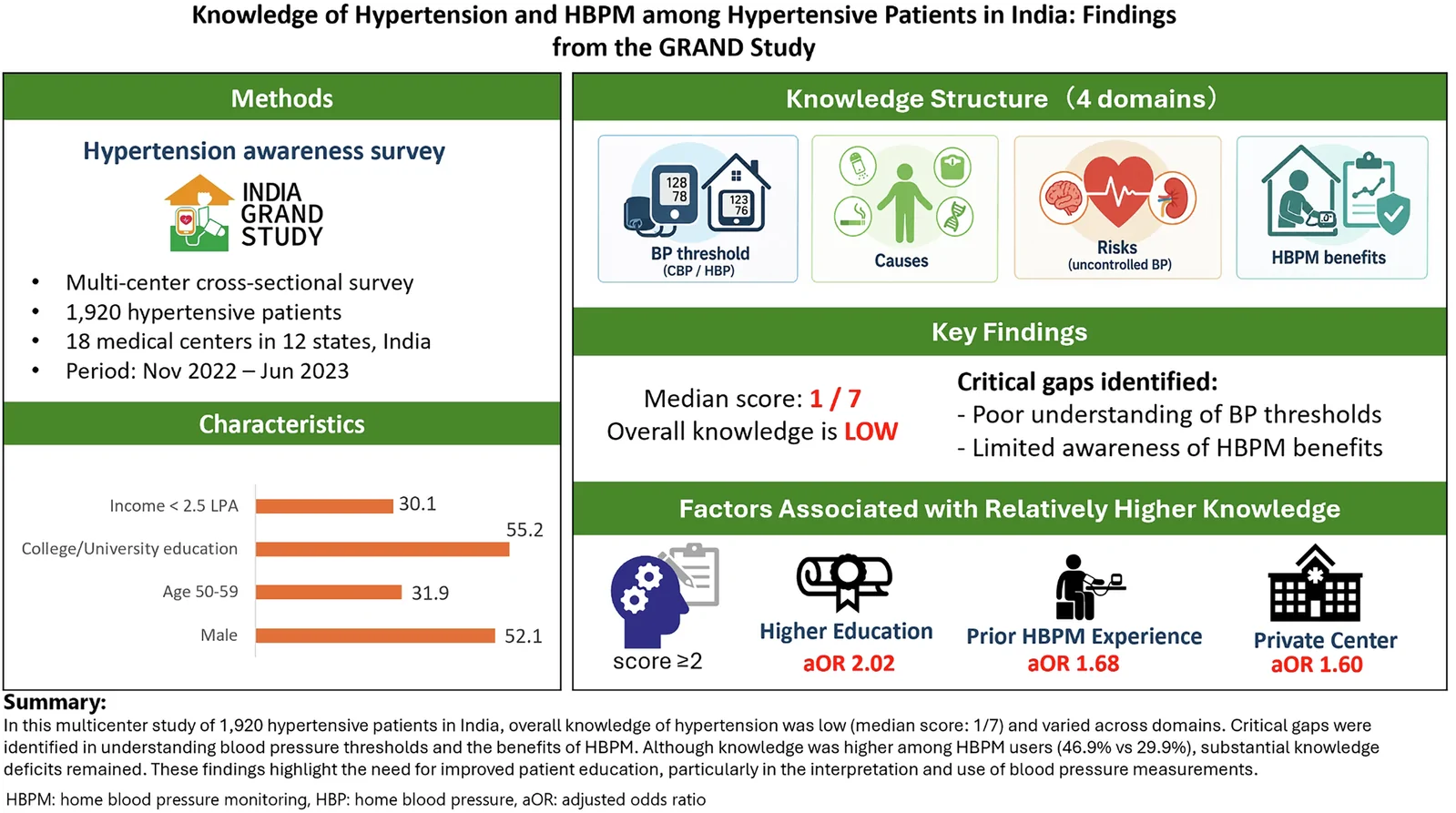

## Introduction

Hypertension is a major risk factor for cardiovascular diseases (CVDs) in India [1]. According to the Global Burden of Disease Study, hypertension accounted for approximately 1.54 million deaths and 38.1 million disability-adjusted life years in 2017 [2]. Despite increasing prevalence, estimated at 30.7% among adults in 2019 [3], blood pressure (BP) control remains suboptimal, with control rates reported as low as 8.5% [4]. Improving hypertension management is therefore a major public health priority in India.

Adequate patient knowledge of hypertension, including its causes, associated risks, and management, is essential for effective disease control. However, previous studies in India have demonstrated insufficient patient awareness, particularly regarding the risks of uncontrolled hypertension such as stroke and cardiovascular disease [5].

Home blood pressure monitoring (HBPM) is widely recommended in clinical guidelines as a useful tool for hypertension diagnosis and management because of its practicality and reliability [6]. Several hypertension guidelines endorse the integration of HBPM into clinical practice [7–9]. Nevertheless, the uptake of HBPM remains limited in many developing settings, including India.

While previous research has indicated an association between hypertension knowledge and BP control, the existing evidence in India is constrained by small sample sizes and limited geographic scope [10, 11]. Notably, evidence regarding knowledge of HBPM among hypertensive patients in India remains limited. Therefore, this study aimed to assess knowledge of hypertension and HBPM among hypertensive patients across multiple regions in India and to identify factors associated with relatively higher knowledge.

Point of view

- Clinical relevance:
  Approximately half of the participants had received antihypertensive treatment for ≥5 years, and more than half had attained a college or university education. Nevertheless, overall knowledge regarding hypertension and HBPM remained low. Although participants with prior HBPM experience were more likely to demonstrate relatively higher knowledge, these findings suggest that promoting HBPM alone is insufficient and should be accompanied by structured patient education.
- Future direction:
  Future prospective studies should determine whether targeted educational interventions can improve patient knowledge, promote the appropriate use of HBPM, and ultimately improve long-term blood pressure control and cardiovascular outcomes. Implementation studies are also warranted to identify effective strategies for integrating patient education and HBPM into routine hypertension care.
- Consideration for the Asian population:
  Many Asian countries share similar challenges in hypertension awareness, patient education, and implementation of HBPM. The findings of this multicenter study from India provide practical insights for strengthening patient education and promoting the appropriate use of HBPM across Asia.

## Materials and methods

### Participants and study design

Participants were derived from the GeogRaphic And socioecoNomic Distribution of real-world Indian data of home blood pressure monitoring (GRAND Study), a multicenter observational study conducted between November 2022 and June 2023 at 18 medical centers across 12 states in India. Eligible participants were adults aged 20–70 years who had been receiving stable antihypertensive medication for at least 3 months. Detailed study methods have been described elsewhere [12].

A total of 1920 participants completed the baseline survey and were included in the present analysis, which focused on patient-reported knowledge related to hypertension and HBPM, including prior HBPM experience. To assess the representativeness of the study population, the regional distribution of participants was compared with national population estimates (Supplementary Table 3).

### Survey instrument

The Baseline Patient Recognition Survey (Visit 1) was administered prior to the initiation of the 7-day HBPM protocol. The questionnaire was designed to assess patient knowledge of hypertension and HBPM.

The survey consisted of 21 items. Questions 1–10 were answered by all participants, while subsequent questions were administered depending on responses to the question regarding prior HBPM experience. Participants were categorized into two groups based on self-reported prior HBPM experience: those with prior HBPM experience and those without prior HBPM experience.

### Assessment of knowledge

Knowledge of hypertension and HBPM was assessed at baseline using a structured questionnaire developed based on previously published studies and refined for the objectives of this study. The selected items covered four domains: (1) understanding of clinic and home blood pressure thresholds, (2) awareness of the causes of hypertension, (3) recognition of risks associated with uncontrolled blood pressure, and (4) awareness of the benefits of HBPM (Supplementary Fig. 1).

A composite knowledge score was constructed based on predefined knowledge-related items (Supplementary Table 1). The scored items included knowledge of clinic and home blood pressure thresholds, causes of hypertension, risks of uncontrolled hypertension, and benefits of HBPM.

Each item was assigned one point if the predefined correctness criteria were met and zero otherwise. The total knowledge score ranged from 0 to 7, with higher scores indicating greater knowledge.

To minimize the potential impact of random responses, several items included distractor options that were not counted in the scoring. Participants were categorized into relatively higher and lower knowledge groups based on the 75th percentile of the total score distribution. In this study, a score of ≥2 corresponded to the threshold for relatively higher knowledge. Sensitivity analyses were additionally performed using a stricter alternative definition of relatively higher knowledge (knowledge score ≥3).

### Statistical analysis

Continuous variables are presented as mean ± standard deviation, and categorical variables as numbers and percentages. Differences between groups were assessed using independent-samples *t*-tests for continuous variables and chi-square tests for categorical variables. Comparisons of knowledge scores between HBPM and non-HBPM groups were additionally assessed using the Mann–Whitney U test due to the non-normal distribution of the knowledge scores.

Multivariable logistic regression analysis was performed to identify factors independently associated with membership in the higher knowledge group (defined as ≥75th percentile of the total knowledge score; cutoff ≥2). The following variables were included in the final model: HBPM use, age (continuous), sex, education level, type of healthcare facility, and epidemiological transition level (ETL). Results are presented as odds ratios (ORs) with 95% confidence intervals (CIs). Sensitivity analyses using an alternative cutoff for relatively higher knowledge (knowledge score ≥3) were additionally performed to assess the robustness of the findings.

All statistical tests were two-sided, and a *p*-value < 0.05 was considered statistically significant. Analyses were conducted using SPSS version 25 (IBM Corp., Armonk, NY, USA).

## Results

### Characteristics of study participants

Baseline characteristics of the study participants stratified by prior HBPM experience are presented in Table 1. A total of 1922 hypertensive patients were enrolled, of whom 1920 (99.9%) provided valid responses and were included in the analysis. Among them, 912 participants (47.5%) reported prior HBPM experience. Prior HBPM experience was used as an exposure variable in subsequent analyses of knowledge scores.

**Table 1:** Baseline demographic, socioeconomic, clinical, and lifestyle characteristics of all study participants by prior HBPM experience (*N* = 1920)

Characteristics	Overall *N* = 1920		HBP measurement *N* = 912 (47.5%)		Non-HBP measurement *N* = 1008 (52.5%)		*P*-value
	*N*	(%)	*N*	(%)	*N*	(%)	
Age							0.005
20–29 years	28	(1.5)	16	(1.8)	12	(1.2)	
30–39 years	202	(10.5)	98	(10.7)	104	(10.3)	
40–49 years	466	(24.3)	243	(26.6)	223	(22.1)	
50–59 years	612	(31.9)	294	(32.2)	318	(31.5)	
60–69 years	557	(29.0)	229	(25.1)	328	(32.5)	
70 years	55	(2.9)	32	(3.5)	23	(2.3)	
Gender							0.616
Male	1001	(52.1)	470	(51.5)	531	(52.7)	
Religion							<0.001
Christian	82	(4.3)	3	(0.3)	79	(7.8)	
Hindu	1687	(87.9)	843	(92.4)	844	(83.7)	
Muslims	115	(6.0)	55	(6.0)	60	(6.0)	
Sikh	36	(1.9)	11	(1.2)	25	(2.5)	
Socio-economic status							<0.001
<2.5 LPA	578	(30.1)	347	(38.0)	231	(22.9)	
2.6–5 LPA	476	(24.8)	173	(19.0)	303	(30.1)	
5.1–10 LPA	299	(15.6)	125	(13.7)	174	(17.3)	
**≥**10.1 LPA	566	(29.5)	267	(29.3)	299	(29.7)	
Marital status							<0.001
Unmarried	47	(2.4)	15	(1.6)	32	(3.2)	
Without children	780	(40.6)	493	(54.1)	287	(28.5)	
With children	1093	(56.9)	404	(44.3)	689	(68.4)	
Education level							<0.001
None	85	(4.4)	21	(2.3)	64	(6.3)	
Primary	182	(9.5)	46	(5.0)	136	(13.5)	
Secondary	218	(11.4)	82	(9.0)	136	(13.5)	
High school	320	(16.7)	142	(15.6)	178	(17.7)	
College/University	1059	(55.2)	595	(65.2)	464	(46.0)	
Others	56	(2.9)	26	(2.9)	30	(3.0)	
Occupation							<0.001
Agricultural workers	66	(3.4)	5	(0.5)	61	(6.1)	
Defense worker	10	(0.5)	3	(0.3)	7	(0.7)	
Housewife	594	(30.9)	292	(32.0)	302	(30.0)	
Office worker	558	(29.1)	292	(32.0)	266	(26.4)	
Retired	146	(7.6)	85	(9.3)	61	(6.1)	
Self-employed	424	(22.1)	194	(21.3)	230	(22.8)	
Student	1	(0.1)	0	(0.0)	1	(0.1)	
Unemployed	71	(3.7)	31	(3.4)	40	(4.0)	
Unskilled worker	50	(2.6)	10	(1.1)	40	(4.0)	
Health insurance							<0.001
Yes	916	(47.7)	366	(40.1)	550	(54.6)	
Duration of hypertension							0.037
<5 years	994	(51.8)	495	(54.3)	499	(49.5)	
≥5 years	926	(48.2)	417	(45.7)	509	(50.5)	
Duration of hypertension therapy							0.002
<5 years	1055	(54.9)	535	(58.7)	520	(51.6)	
≥5 years	865	(45.1)	377	(41.3)	488	(48.4)	
Hyperlipidemia							<0.001
Yes	959	(49.9)	594	(65.1)	365	(36.2)	
Diabetes mellitus							<0.001
Yes	756	(39.4)	318	(34.9)	438	(43.5)	
Chronic kidney disease							0.047
Yes	17	(0.9)	4	(0.4)	13	(1.3)	
Comorbidity							<0.001
0	562	(29.3)	178	(19.5)	384	(38.1)	
1	956	(49.8)	533	(58.4)	423	(42.0)	
2	371	(19.3)	184	(20.2)	187	(18.6)	
≥3	31	(1.6)	17	(1.9)	14	(1.4)	
Sleep pattern							0.001
Less than 5 h	138	(7.2)	46	(5.0)	92	(9.1)	
6–8 h	1576	(82.1)	778	(85.3)	798	(79.2)	
More than 8 h	206	(10.7)	88	(9.6)	118	(11.7)	
Physical activity							<0.001
Low (<30 min a day)	571	(29.7)	254	(27.9)	317	(31.4)	
Medium (30–60 mins a day)	826	(43.0)	337	(37.0)	489	(48.5)	
High (>60 min a day)	523	(27.2)	321	(35.2)	202	(20.0)	
Diet habit							<0.001
Vegetarian	844	(44.0)	513	(56.3)	331	(32.8)	
Non-vegetarian	1076	(56.0)	399	(43.8)	677	(67.2)	
Health center type							
Public	476	(24.8)	146	(16.0)	330	(32.7)	<0.001
Private	1444	(75.2)	766	(84.0)	678	(67.3)	

Values are presented as number (*n*) and percentage (%). Comparisons between participants with and without prior HBPM experience were performed using chi-square tests for categorical variables. A two-sided *P* value < 0.05 was considered statistically significant

Significant differences were observed for age, religion, socioeconomic status, marital status, education level, occupation, health insurance coverage, duration of hypertension and therapy, presence of comorbidities, sleep pattern, physical activity, diet habit, and type of health facility

*HBPM* home blood pressure monitoring, *LPA* Lakhs Per Annum; Comorbidity includes hyperlipidemia, diabetes mellitus, liver disease, chronic kidney disease, thoracic aortic aneurysm, carotid artery disease, and atrial fibrillation

Overall, the majority of participants (85.2%) were aged between 40 and 69 years, and more than half (55.2%) had attained a college or university level of education. Income distribution was relatively balanced, with approximately 30% of participants in both the lowest and highest income categories. Health insurance coverage was reported by 47.7% of participants. In terms of clinical characteristics, approximately 70% of participants had at least one comorbidity in addition to hypertension, and the prevalence of diabetes mellitus was 39.4%.

When stratified by prior HBPM experience, several differences were observed. Among participants with prior HBPM experience, the lowest income group (<2.5 LPA) was the most common (38.0%). Health insurance coverage was lower among participants with prior HBPM experience (40.0%) compared with those without prior HBPM experience. In contrast, diabetes mellitus was more prevalent among participants without prior HBPM experience than among those with prior HBPM experience (43.5% vs. 34.9%, *p* < 0.001). Participants with prior HBPM experience were also more likely to have at least one comorbidity. Overall, despite these differences, many demographic and clinical characteristics were broadly comparable between the two groups. The regional distribution of participants showed some variation compared with national population estimates, with certain regions being over- or under-represented (Supplementary Table 3).

### Knowledge scores according to prior HBPM experience

Table 2 summarizes knowledge scores according to prior HBPM experience. The median knowledge score was low in both groups (median [interquartile range]: 1 [0–2]). However, the proportion of participants in the relatively higher knowledge group (≥75th percentile of the score distribution; cutoff ≥2) was significantly higher among participants with prior HBPM experience than among those without prior HBPM experience (46.9% vs. 29.9%, *p* < 0.001). The distribution of total knowledge scores demonstrated that the majority of participants had low knowledge scores (Supplementary Table 6).

**Table 2:** Knowledge score according to prior HBPM status

Variable	Overall	HBPM	Non-HBPM	*p*-value
	*N* = 1920	*N* = 912	*N* = 1008	
Knowledge score, median (interquartile range)	1 (0–2)	1 (0–2)	1 (0–2)	<0.001
Higher knowledge (≥75th percentile)	729 (38.0%)	428 (46.9%)	301 (29.9%)	<0.001

Data are presented as median (interquartile range) or *n* (%). *P* values for knowledge scores were calculated using the Mann–Whitney U test. Categorical variables were compared using the chi-square test

The higher knowledge group was defined as participants with knowledge scores at or above the 75th percentile of the score distribution (cutoff ≥ 2). The total knowledge score ranged from 0 to 7, with higher scores indicating greater knowledge. A two-sided *P* value < 0.05 was considered statistically significant

Supplementary Table 2 presents item-level knowledge of hypertension according to prior HBPM experience. Knowledge regarding clinic and home blood pressure thresholds was generally limited in both groups. Participants with prior HBPM experience demonstrated better knowledge across several domains, including awareness of clinic blood pressure systolic and diastolic cut-off values, causes of hypertension, risks associated with uncontrolled hypertension, and benefits of HBPM. In particular, knowledge of clinic blood pressure diastolic cut-off values was substantially higher among participants with prior HBPM experience than among those without prior HBPM experience (39.4% vs. 14.7%, *p* < 0.001). Participants with prior HBPM experience were also more likely to recognize lifestyle-related risk factors and major cardiovascular complications.

Sensitivity analyses using a stricter alternative definition of the relatively higher knowledge group (knowledge score ≥3) showed similar results, with participants with prior HBPM experience more likely to belong to the relatively higher knowledge group than those without prior HBPM experience (20.9% vs. 10.5%, *p* < 0.001) (Supplementary Table 4). Sensitivity analyses using multivariable logistic regression with the alternative cutoff demonstrated generally consistent findings, with prior HBPM experience, higher education level, private center type, and high ETL remaining significantly associated with relatively higher knowledge (Supplementary Table 5).

### Comparison of characteristics according to knowledge level

Table 3 summarizes the comparison of participant characteristics according to knowledge level. Participants with relatively higher knowledge were significantly younger than those with low knowledge (51.6 ± 10.8 vs. 53.6 ± 10.3 years, *p* < 0.001). The proportion of participants with prior HBPM experience was significantly higher in the relatively higher knowledge group compared with the low knowledge group (58.7% vs. 40.6%, *p* < 0.001). Similarly, participants with higher education levels were more likely to belong to the relatively higher knowledge group (68.0% vs. 47.3%, *p* < 0.001). There were no significant differences in sex (54.3% vs. 50.8%, *p* = 0.134) or duration of hypertension ≥5 years (45.8% vs. 49.7%, *p* = 0.098) between the groups.

**Table 3:** Comparison of characteristics according to higher knowledge group status

Variables	Overall	Low knowledge	Higher knowledge	*p*-value
	*N* = 1920	*N* = 1191	N = 729	
Age, mean ± SD	52.84 ± 10.54	53.58 ± 10.31	51.62 ± 10.80	<0.001
Male, *n* (%)	919 (47.9%)	605 (50.8%)	396 (54.3%)	0.134
High education level, *n* (%)	1059 (55.2%)	563 (47.3%)	496 (68.0%)	<0.001
Duration of HT ≥ 5 years, *n* (%)	926 (48.2%)	592 (49.7%)	334 (45.8%)	0.098
Prior HBPM experience, *n* (%)	912 (47.5%)	484 (40.6%)	428 (58.7%)	<0.001

Data are presented as mean ± SD or *n* (%). Comparisons were performed using the independent-samples *t*-test for continuous variables and the chi-square test for categorical variables

The higher knowledge group was defined as participants with knowledge scores at or above the 75th percentile of the score distribution (cutoff ≥ 2). A two-sided *P* value < 0.05 was considered statistically significant

*HT* hypertension, *HBPM* home blood pressure monitoring, *SD* standard deviation

### Factors independently associated with relatively higher knowledge

Table 4 presents the results of the multivariable logistic regression analysis. Prior HBPM experience was independently associated with relatively higher knowledge (adjusted odds ratio [aOR] 1.68, 95% confidence interval [CI] 1.36–2.08, *p* < 0.001). Higher education level was also significantly associated with relatively higher knowledge (aOR 2.02, 95% CI 1.64–2.48, *p* < 0.001). Age was inversely associated with relatively higher knowledge, although the effect size was small (aOR 0.99 per year, 95% CI 0.98–1.00, *p* = 0.041). Private center type was significantly associated with relatively higher knowledge (aOR 1.60, 95% CI 1.24–2.07, *p* < 0.001). In contrast, sex (aOR 0.97, 95% CI 0.80–1.18, *p* = 0.739) and ETL (aOR 0.94, 95% CI 0.75–1.19, *p* = 0.619) were not significantly associated with relatively higher knowledge.

**Table 4:** Factors associated with relatively higher knowledge (multivariable logistic regression analysis)

Variables		aOR	95% CI		*P* value
			Lower	Upper	
HBP measurement	Prior HBPM (vs No HBPM)	1.679	1.356	2.078	<0.001
Age	Age (per year)	0.990	0.981	1.000	0.041
Education level	High education (vs Low education)	2.017	1.642	2.478	<0.001
Sex	Female (vs Male)	0.967	0.796	1.176	0.739
Center type	Private (vs Public)	1.604	1.241	2.074	<0.001
ETL	Low ETL (vs High ETL)	0.944	0.752	1.185	0.619

Multivariable logistic regression analysis was performed to identify factors associated with relatively higher knowledge. Variables included in the model were prior HBPM experience, age, sex, education level, center type, and epidemiological transition level (ETL). Age was entered as a continuous variable (per 1-year increase). The relatively higher knowledge group was defined as participants with knowledge scores at or above the 75th percentile of the score distribution (cutoff ≥ 2). Adjusted odds ratios (aORs) and 95% confidence intervals (CIs) are presented

*HBP* home blood pressure, *HBPM* home blood pressure monitoring, *aOR* adjusted odds ratio, *CI* confidence interval, *ETL* epidemiological transition level

In sensitivity analyses using multivariable logistic regression with a stricter alternative definition of relatively higher knowledge (knowledge score ≥3), prior HBPM experience, higher education level, private center type, and high ETL remained significantly associated with relatively higher knowledge (Supplementary Table 5). Age and sex were not significantly associated with relatively higher knowledge in the sensitivity analysis.

## Discussion

In this large, multicenter study of hypertensive patients in India, knowledge of hypertension and HBPM was generally low and varied across domains. Notably, nearly half of the participants (47.5%) reported prior HBPM experience before study participation. Despite this relatively high level of uptake, overall knowledge remained limited, particularly regarding BP thresholds and the benefits of HBPM. This suggests that HBPM may often be practiced without adequate understanding, indicating a gap between adoption and effective use.

Item-level analyses (Supplementary Table 2) revealed that understanding of BP thresholds, particularly for HBP, and awareness of the benefits of HBPM were notably limited, whereas recognition of lifestyle-related risk factors was relatively higher. While awareness of certain risk factors, such as high salt intake, was relatively good, misconceptions were also observed, including incorrect attribution to unrelated causes. In addition, awareness of specific complications was suboptimal, and a proportion of participants reported not knowing the risks of uncontrolled hypertension. These findings suggest that knowledge deficits are not uniform but are concentrated in clinically important domains, particularly those related to the interpretation and application of BP measurements.

Participants with prior HBPM experience demonstrated better understanding of home BP thresholds, lifestyle-related risk factors, and major cardiovascular complications. This finding is consistent with previous studies showing that patient knowledge and self-management behaviors are associated with BP control [13, 14]. However, prior studies in India have also reported a discordance between knowledge and practice. In a multicentric study, knowledge of HBPM was relatively low (~40%), whereas practice scores were higher, suggesting that patients may perform BP measurements without fully understanding their principles. Moreover, most participants reported learning HBPM independently, with only a minority receiving formal training from healthcare professionals [15]. In addition, findings from the Asia HBPM Survey indicate that although many physicians recommend HBPM, relatively few provide detailed guidance on its appropriate use [16]. Taken together, these findings suggest that HBPM is often implemented without sufficient patient education, which may limit its effectiveness in improving hypertension management.

In our study, participants with prior HBPM experience demonstrated higher knowledge scores and were more frequently classified as belonging to the relatively higher knowledge group compared with those without prior HBPM experience. This association remained significant even after adjusting for variables such as age, sex, education level, type of healthcare facility, and ETL. However, it is important to note that causality cannot be inferred due to the cross-sectional nature of the study design. In addition, although statistically significant differences were observed between participants with and without prior HBPM experience, the absolute differences in knowledge scores were modest, and the median knowledge scores were similar between groups. Therefore, these findings should be interpreted as indicating relatively higher knowledge among participants with prior HBPM experience rather than sufficient knowledge overall. Although the median knowledge score was similarly low in both groups (median [interquartile range]: 1 [0–2]), the proportion of participants classified as having relatively higher knowledge was substantially greater among participants with prior HBPM experience than among those without prior HBPM experience (46.9% vs. 29.9%). Importantly, similar findings were observed in sensitivity analyses using a stricter cutoff (knowledge score ≥3), in which participants with prior HBPM experience remained approximately twice as likely to belong to the relatively higher knowledge group compared with those without prior HBPM experience (20.9% vs. 10.5%). These findings further support the robustness of the observed association between prior HBPM experience and relatively higher knowledge.

Education level was the strongest factor associated with relatively higher knowledge, with higher education linked to approximately a twofold increase in the likelihood of belonging to the relatively higher knowledge group. This is consistent with previous studies demonstrating that patient knowledge and self-management are associated with BP control [12, 13]. Notably, the association between prior HBPM experience and relatively higher knowledge remained significant after adjustment for education level, suggesting that engagement with BP monitoring may be associated with greater awareness, although the direction of this relationship cannot be determined.

Age showed a modest inverse association with relatively higher knowledge, indicating slightly higher knowledge among younger individuals, possibly reflecting differences in access to health information and familiarity with self-monitoring practices. In contrast, sex and duration of hypertension were not associated with knowledge, suggesting that knowledge does not necessarily improve over time without active educational interventions.

Healthcare system factors also contributed to differences in knowledge. Type of healthcare facility was significantly associated with knowledge, indicating that variations in clinical practice and patient education across healthcare settings may influence patient understanding. In contrast, ETL was not associated with knowledge, suggesting that individual-level factors may be more important than regional characteristics.

The interpretation of income-related findings requires caution. HBPM users included participants from lower socioeconomic groups, and this should not be interpreted as evidence that lower income directly promotes HBPM use. Rather, this finding may reflect differences in healthcare-seeking behavior, healthcare access, or prioritization of medical expenditure. Similar observations have been reported in India, where healthcare utilization does not always correspond directly with income level [17]. These findings suggest that the relationship between socioeconomic status and HBPM use is complex and warrants further investigation.

In India, various initiatives by governmental agencies and professional societies have aimed to improve awareness of hypertension and its management. However, systematic dissemination of knowledge regarding proper BP measurement and the role of HBPM remains limited. This context may help explain the relatively low level of knowledge observed in this study.

Future efforts should focus on strengthening patient education on BP thresholds, the risks of uncontrolled hypertension, and proper BP measurement techniques. Practical interventions, such as the development and distribution of hypertension patient kits incorporating BP diaries and educational materials, may support patient understanding and self-management. In addition, enhancing guidance from healthcare professionals is essential to ensure the appropriate use of HBPM. Strengthening education among healthcare providers, including medical and nursing trainees, may further improve knowledge dissemination and contribute to better long-term hypertension management. From a clinical and public health perspective, these findings highlight the importance of a comprehensive approach that combines patient education with the appropriate use of HBPM. These findings remained consistent in sensitivity analyses using a stricter definition of the higher knowledge group (knowledge score ≥3), supporting the robustness of the observed associations.

### Perspective of Asia

India has one of the largest populations of patients with hypertension in Asia, and improving hypertension control remains a major public health priority. Although HBPM is increasingly recommended in international and regional hypertension guidelines, patient knowledge regarding hypertension and the appropriate use of HBPM remains limited. The present findings highlight important knowledge gaps in BP thresholds, the risks of uncontrolled hypertension, and the benefits of HBPM among treated hypertensive patients. These findings are likely to be relevant not only to India but also to many Asian countries facing similar challenges in hypertension awareness, patient education, and implementation of HBPM. Strengthening patient education together with appropriate guidance from healthcare professionals may facilitate more effective use of HBPM and contribute to improved hypertension management across Asia.

### Study limitations

Several limitations should be acknowledged. First, this study employed a cross-sectional design, which precludes causal inference; therefore, it remains unclear whether HBPM leads to higher knowledge or whether individuals with greater knowledge are more likely to adopt HBPM. Second, knowledge of hypertension was assessed using a questionnaire that has not been internationally standardized. Although the instrument was developed based on previously published studies and tailored to capture key knowledge domains, it may not fully reflect all aspects of hypertension awareness. Third, HBPM use was self-reported and may be subject to recall bias. Fourth, the study population may be subject to selection bias. Participants were predominantly recruited from private healthcare facilities, which account for a large proportion of healthcare delivery in India, but may not fully represent patients receiving care in public settings. In addition, the educational level of participants was higher than national estimates, and some individuals classified as low-income in this study may correspond to middle-income groups in the broader Indian population. Finally, although participants were recruited from 12 states across India, the sample size remains limited relative to the overall population. Therefore, the findings should be interpreted within the context of the study population and may not be fully generalizable to the entire Indian population.

## Conclusion

This study demonstrated that knowledge of hypertension and HBPM among patients in India was generally low and varied across domains. In particular, important gaps were identified in understanding BP thresholds and the benefits of HBPM. Although relatively higher knowledge was observed among participants with prior HBPM experience, overall knowledge remained insufficient. Improving hypertension management requires enhancing patients’ understanding of the risks of hypertension and the importance of BP control, as well as promoting the appropriate use of HBPM based on a clear understanding of its benefits.

## Supplementary information

Supplementary Table 1

Supplementary Table 2

Supplementary Table 3

Supplementary Table 4

Supplementary Table 5

Supplementary Table 6

Supplementary Information
