# Supplementary material for: Knowledge of hypertension and understanding of home blood pressure monitoring among hypertensive patients in India: results from the GRAND Study
Source: Hypertens Res. 2026 Aug 21;49(11):2936–45. doi: 10.1038/s41440-026-02763-w (PMC13736692; doi:10.1038/s41440-026-02763-w)
Supplement: Supplementary file 1 [file 41440_2026_2763_MOESM7_ESM.pdf]

Supplementary Figure 1. Structure of knowledge assessment for hypertension and HBPM

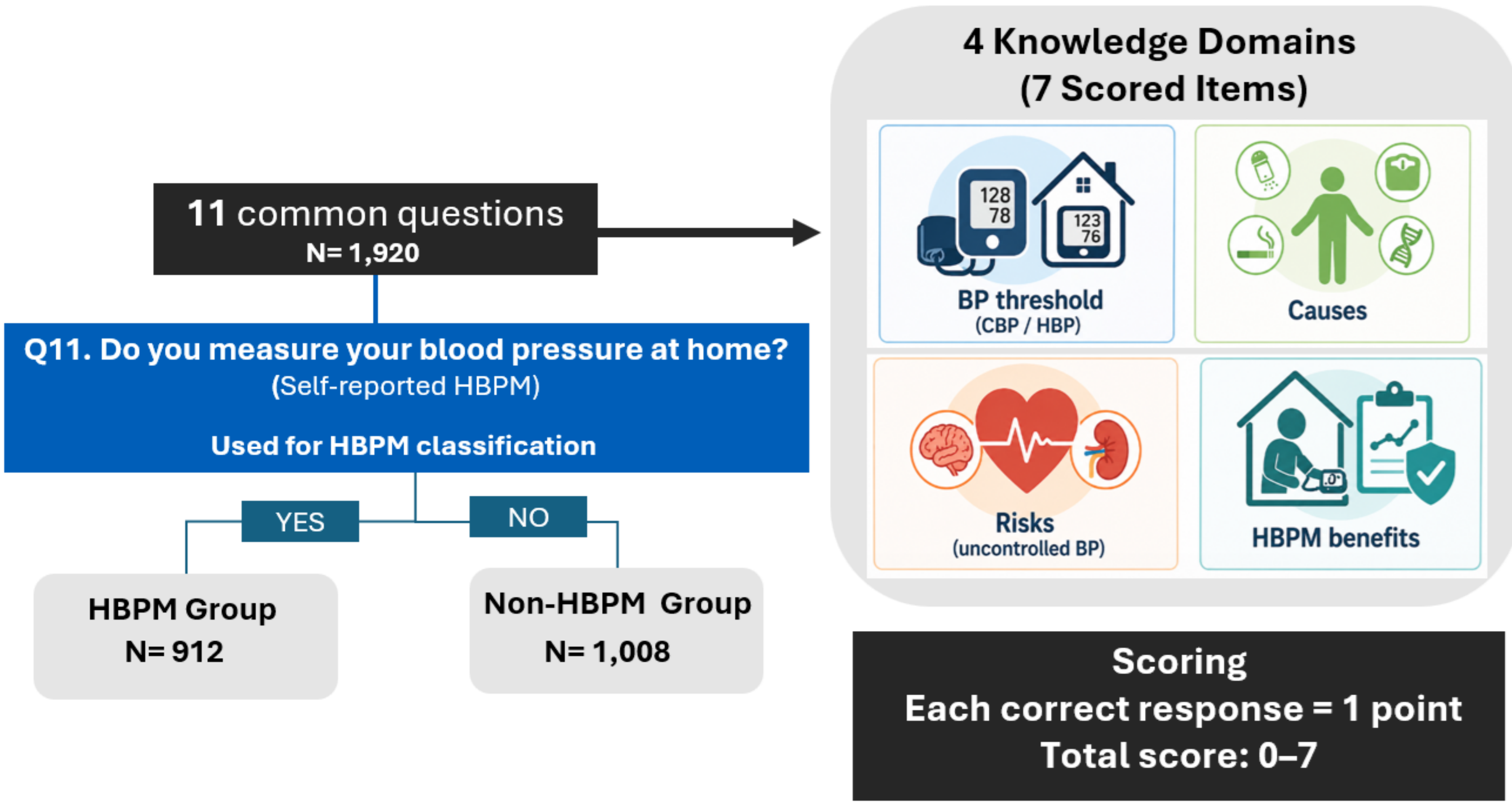

Participants were classified as HBPM users or non-users based on self-reported home BP measurement. A total of 11 questions were included in the survey, of which 7 items were used to construct the knowledge score across four domains: BP thresholds, causes, risks of uncontrolled BP, and HBPM benefits. These domains were used to calculate the overall knowledge score. Each correct response was assigned 1 point, and the total score ranged from 0 to 7.
