# Supplementary material for: Knowledge of hypertension and understanding of home blood pressure monitoring among hypertensive patients in India: results from the GRAND Study
Source: Hypertens Res. 2026 Aug 21;49(11):2936–45. doi: 10.1038/s41440-026-02763-w (PMC13736692; doi:10.1038/s41440-026-02763-w)
Supplement: Supplementary file 2 [file 41440_2026_2763_MOESM1_ESM.docx]

**Supplementary Table 1. Knowledge Score Domains, Scoring Items, and Predefined Scoring Criteria**

| **Domain** | **Question No.** | **Question** | **Correct Answer / Scoring Criteria** | **Score** |
| --- | --- | --- | --- | --- |
| BP threshold | Q1-1 | Knows clinic SBP cut-off | Correct if answered 140 mmHg | 1 |
| BP threshold | Q1-2 | Knows clinic DBP cut-off | Correct if answered 90 mmHg | 1 |
| BP threshold | Q1-3 | Knows home SBP cut-off | Correct if answered 135 mmHg | 1 |
| BP threshold | Q1-4 | Knows home DBP cut-off | Correct if answered 85 mmHg | 1 |
| Causes | Q3 | Knows ≥4 causes of hypertension | ≥4 correct selections | 1 |
| Risks | Q4 | Knows ≥2 risks of uncontrolled hypertension | ≥2 correct selections | 1 |
| HBPM benefits | Q8 | Knows ≥4 benefits of HBPM | ≥4 correct selections | 1 |

*Each scored item contributed 1 point to the composite knowledge score (range: 0–7). Participants received 1 point only when the predefined correctness criteria were met; otherwise, 0 points were assigned.*
