# Supplementary material for: Knowledge of hypertension and understanding of home blood pressure monitoring among hypertensive patients in India: results from the GRAND Study
Source: Hypertens Res. 2026 Aug 21;49(11):2936–45. doi: 10.1038/s41440-026-02763-w (PMC13736692; doi:10.1038/s41440-026-02763-w)
Supplement: Supplementary file 3 [file 41440_2026_2763_MOESM2_ESM.docx]

**Supplementary Table 2. Item-level Knowledge of Hypertension According to Prior HBPM Experience**

| **Question** | | **Overall N= 1,920** | **HBPM N= 912** | **Non HBPM N= 1,008** | **p-value** |
| --- | --- | --- | --- | --- | --- |
| **From your understanding, what is considered high blood pressure value (hypertension)?** | |  |  |  |  |
| * | Knows CBP systolic cut-off | 15.3% | 19.1% | 11.9% | <0.001 |
| * | Knows CBP diastolic cut-off | 26.4% | 16.2% | 14.7% | <0.001 |
| * | Knows HBP systolic cut-off | 7.0% | 13.8% | 0.8% | <0.001 |
| * | Knows HBP diastolic cut-off | 1.5% | 2.4% | 0.6% | 0.001 |
| **Are you aware of the causes of hypertension?** | |  |  |  |  |
| * | Diet rich in salt | 77.4% | 73.0% | 81.3% | <0.001 |
| * | High alcohol consumption | 15.1% | 16.7% | 13.7% | 0.069 |
| * | Smoking/chewing tobacco | 35.4% | 29.1% | 41.1% | <0.001 |
| * | Obesity | 34.3% | 38.9% | 30.1% | <0.001 |
| * | Lack of physical activity | 31.0% | 37.5% | 25.2% | <0.001 |
| * | Stress | 53.3% | 57.2% | 49.7% | 0.001 |
| * | Family history of high blood pressure | 19.4% | 20.2% | 18.8% | 0.430 |
| * | Association with other health conditions (Chronic kidney disease…etc.) | 5.3% | 4.2% | 6.3% | 0.041 |
|  | Vaccination | 1.7% | 2.0% | 1.4% | 0.318 |
|  | Blood type | 10.3% | 8.8% | 11.7% | 0.035 |
| **Are you aware about the health risk of uncontrolled hypertension?** | |  |  |  |  |
| **Please choose from the following options:** | |  |  |  |  |
|  | Cancer | 7.1% | 9.2% | 5.3% | 0.001 |
| * | Organ Damage | 37.7% | 41.6% | 34.2% | 0.035 |
| * | Heart Attack/ Heart Stroke | 70.4% | 73.8% | 67.4% | <0.001 |
| * | Heart Failure | 47.6% | 54.4% | 41.5% | 0.003 |
|  | Don’t know | 9.6% | 7.5% | 11.5% | 0.003 |
| * | Others | 0.0% | 0.0% | 0.0% | NA |
| **In your opinion, what are the benefits of home blood pressure monitoring?** | |  |  |  |  |
| * | To realize the difference between my clinic blood pressure and usual blood pressure | 25.8% | 37.3% | 15.5% | <0.001 |
| * | Helps to know if my hypertension medication is effective | 38.1% | 45.5% | 31.3% | <0.001 |
| * | Regular monitoring helps to manage my other health conditions | 49.2% | 69.0% | 31.3% | <0.001 |
| * | Good practice for early warning and prevent CVD (e.g., stroke, heart attacks) | 33.9% | 46.6% | 22.4% | <0.001 |
| * | Adjust lifestyle habits for better health | 28.5% | 38.3% | 19.6% | <0.001 |
| * | I can measure more frequently my blood pressure at home | 15.5% | 20.1% | 11.4% | <0.001 |
|  | I can save time going to the clinic/pharmacy for only measuring my blood pressure | 10.3% | 8.1% | 12.3% | 0.003 |
|  | I do not think there are any benefits of home blood pressure monitoring | 5.0% | 2.1% | 7.6% | <0.001 |

*Data are presented as percentages. Participants were asked to select applicable responses for each knowledge item. Comparisons between HBPM and non-HBPM groups were performed using the chi-square test. A two-sided P value < 0.05 was considered statistically significant.*

*Items marked with an asterisk (*) were included in the calculation of the knowledge score.*

*Correct knowledge items included awareness of blood pressure cut-off values, modifiable risk factors (e.g., high salt intake, obesity, physical inactivity), and complications of uncontrolled hypertension (e.g., stroke, heart failure). Some items (e.g., vaccination, blood type) were included to assess misconceptions.*

CBP, clinic blood pressure; HBP, home blood pressure; HBPM, home blood pressure monitoring.
