# Supplementary material for: Knowledge of hypertension and understanding of home blood pressure monitoring among hypertensive patients in India: results from the GRAND Study
Source: Hypertens Res. 2026 Aug 21;49(11):2936–45. doi: 10.1038/s41440-026-02763-w (PMC13736692; doi:10.1038/s41440-026-02763-w)
Supplement: Supplementary file 4 [file 41440_2026_2763_MOESM3_ESM.docx]

**Supplementary Table 3. Comparison of Regional Distribution of Participants in The GRAND Study and Estimated Population Distribution in India (2021)**

|  | **Region** | | **GRAND Study** | | | | **Estimated Population 2021** | | | | **Over/Under-representation** | |
| --- | --- | --- | --- | --- | --- | --- | --- | --- | --- | --- | --- | --- |
|  |  |  | **N** | | **(%)** | | **N** | | **(%)** | |  |  |
| Central India | | 661 | | (34.4) | | 180,287,800 | | (36.2) | | Slightly underrepresented | |  |
| East | | 275 | | (14.3) | | 86,721,000 | | (17.4) | | Underrepresented | |  |
| North Himalaya | | 78 | | (4.1) | | 6,211,772 | | (1.2) | | Overrepresented | |  |
| North | | 250 | | (13.0) | | 38,438,600 | | (7.7) | | Overrepresented | |  |
| North-East | | 72 | | (3.8) | | 1,486,304 | | (0.3) | | Overrepresented | |  |
| South | | 436 | | (22.7) | | 95,355,600 | | (19.1) | | Well to slightly overrepresented | |  |
| West | | 148 | | (7.7) | | 89,618,800 | | (18.0) | | Clearly underrepresented | |  |

*To evaluate the representativeness of the study population, the regional distribution of participants in the GRAND Study was compared with the estimated population distribution of adults aged 20–70 years across the participating states in India. Population estimates were based on the 2021 projections from the National Commission on Population.*

*For two states (Meghalaya and Uttarakhand) where age-specific data were not available, estimates were derived using age distribution data from the 2011 Census and applied to the projected 2021 population. The 70-year-old population was approximated by dividing the population aged 70–74 years by five.*

*Over- or under-representation was classified based on the absolute difference between participant proportions and estimated population proportions: differences <3% were considered slight, 3–5% as moderate, and >5% as substantial over- or under-representation.*
