# Supplementary material for: Knowledge of hypertension and understanding of home blood pressure monitoring among hypertensive patients in India: results from the GRAND Study
Source: Hypertens Res. 2026 Aug 21;49(11):2936–45. doi: 10.1038/s41440-026-02763-w (PMC13736692; doi:10.1038/s41440-026-02763-w)
Supplement: Supplementary file 5 [file 41440_2026_2763_MOESM4_ESM.docx]

**Supplementary Table 4. Sensitivity Analysis Using an Alternative Definition of Higher Knowledge (Knowledge Score ≥3)**

| **Variable** | **Overall** | | **HBPM** | | **Non-HBPM** | | **p-value** |
| --- | --- | --- | --- | --- | --- | --- | --- |
|  | **N=1920** | | **N=912** | | **N=1,008** | |  |
|  | **n** | **(%)** | **n** | **(%)** | **n** | **(%)** |  |
| Knowledge score ≥3 | 297 | (15.5) | 191 | (20.9) | 106 | (10.5) | <0.001 |

*Higher knowledge in this sensitivity analysis was defined as a knowledge score ≥3.*

*P values were calculated using the chi-square test.*
