# Supplementary material for: Knowledge of hypertension and understanding of home blood pressure monitoring among hypertensive patients in India: results from the GRAND Study
Source: Hypertens Res. 2026 Aug 21;49(11):2936–45. doi: 10.1038/s41440-026-02763-w (PMC13736692; doi:10.1038/s41440-026-02763-w)
Supplement: Supplementary file 6 [file 41440_2026_2763_MOESM5_ESM.docx]

**Supplementary Table 5. Sensitivity Analysis Using an Alternative Definition of Relatively Higher Knowledge (Knowledge Score ≥3): Multivariable Logistic Regression Analysis**

| **Variables** | | **aOR** | **95% C.I.for EXP(B)** | | **P value** |
| --- | --- | --- | --- | --- | --- |
|  |  |  | **Lower** | **Upper** |  |
| HBP measurement | Prior HBPM (vs No HBPM) | 2.378 | 1.777 | 3.181 | <0.001 |
| Age | Age (per year) | 1.006 | 0.994 | 1.019 | 0.315 |
| Education Level | High education (vs Low education) | 2.775 | 2.060 | 3.739 | <0.001 |
| Sex | Female (vs Male) | 1.167 | 0.899 | 1.516 | 0.245 |
| Center type | Private (vs Public) | 1.489 | 1.017 | 2.178 | 0.041 |
| ETL | High ETL (vs Low ETL) | 2.033 | 1.508 | 2.741 | <0.001 |

*Multivariable logistic regression analysis was performed to identify factors associated with relatively higher knowledge. Variables included in the model were prior HBPM experience, age, sex, education level, center type, and epidemiological transition level (ETL). Age was entered as a continuous variable (per 1-year increase). The relatively higher knowledge group was defined using a stricter alternative cutoff (knowledge score ≥3).*

HBP, home blood pressure; HBPM, home blood pressure monitoring; aOR, adjusted odds ratio; CI, confidence interval; ETL, epidemiological transition level.
