# Supplementary material for: Knowledge of hypertension and understanding of home blood pressure monitoring among hypertensive patients in India: results from the GRAND Study
Source: Hypertens Res. 2026 Aug 21;49(11):2936–45. doi: 10.1038/s41440-026-02763-w (PMC13736692; doi:10.1038/s41440-026-02763-w)
Supplement: Supplementary file 7 [file 41440_2026_2763_MOESM6_ESM.docx]

**Supplementary Table 6. Distribution of Total Knowledge Scores**

| **Knowledge score** | **Overall** | | **HBPM** | | **Non-HBPM** | | **p-value** |
| --- | --- | --- | --- | --- | --- | --- | --- |
|  | **N=1920** | | **N=912** | | **N=1,008** | |  |
|  | **n** | **(%)** | **n** | **(%)** | **n** | **(%)** |  |
| **0** | 618 | (32.2) | 240 | (26.3) | 378 | (37.5) | **<0.001** |
| **1** | 573 | (29.8) | 244 | (26.8) | 329 | (32.6) |  |
| **2** | 432 | (22.5) | 237 | (26.0) | 195 | (19.3) |  |
| **3** | 161 | (8.4) | 121 | (13.3) | 40 | (4.0) |  |
| **4** | 68 | (3.5) | 34 | (3.7) | 34 | (3.4) |  |
| **5** | 61 | (3.2) | 31 | (3.4) | 30 | (3.0) |  |
| **6** | 6 | (0.3) | 4 | (0.4) | 2 | (0.2) |  |
| **7** | 1 | (0.1) | 1 | (0.1) | 0 | (0.0) |  |

*Data are presented as n (%). P values were calculated using the chi-square test. The total knowledge score ranged from 0 to 7, with higher scores indicating greater knowledge.*

*HBPM, home blood pressure monitoring.*
